# Bile Microbiology and Risk Factors Associated with Antibiotic Resistance Patterns in Patients Taken to Laparoscopic Cholecystectomy: A Prospective Cohort Study

**DOI:** 10.3390/antibiotics15080717

**Published:** 2026-07-24

**Authors:** Isabella Van-Londoño, Camilo Ramírez-Giraldo, Samir Moreno-Martinez, Carlos Rodriguez-Barbosa, Maria Gabriela Robayo-Romero, Eliana Maldonado, Susana Rojas López, Andrés Isaza-Restrepo

**Affiliations:** 1 Hospital Universitario Mayor-Méderi, Bogotá 111411, Colombia; ramirezgiraldocamilo@gmail.com (C.R.-G.); doctoraeliana21@hotmail.com (E.M.); susana.rojas@mederi.com.co (S.R.L.); andres.isaza@urosario.edu.co (A.I.-R.); 2 Grupo de Investigación Clínica, Universidad del Rosario, Bogotá 110221, Colombia; samir.moreno@urosario.edu.co (S.M.-M.); rodriguezcarlos0303@gmail.com (C.R.-B.); mariag.robayo@urosario.edu.co (M.G.R.-R.)

**Keywords:** cholecystitis, cholecystectomy, antibiotics, microbiology, bile

## Abstract

**Objectives**: To evaluate bile culture microbiology and factors associated with resistance patterns in patients taken to laparoscopic cholecystectomy to better guide empiric antibiotic therapy. **Methods**: Prospective cohort study with a logistic regression model in a middle-income public hospital network of two institutions. Inclusion criteria were patients taken to laparoscopic cholecystectomy over 18 years of age due to benign biliary disease without other concomitant surgical procedures taken to bile culture and antibiogram testing to evaluate bile culture positivity considered as a “resistant pattern”. **Results**: 226 cultures tested positive for at least one microorganism, and 218 were included in the study. Overall, bile cultures classified as resistant were associated with age, comorbidities, acute signs of cholecystitis, and longer antibiotic therapy. Bile microorganisms found consisted mostly of *Enterobacteriaceae*. In the logistic regression model, previous ERCP, Charlson comorbidity index and duration of antibiotic therapy yielded as statistically significant (p 0.03, p 0.003 and 0.004, respectively) for presenting resistant patterns. **Conclusions**: Patients taken to laparoscopic cholecystectomy have a higher probability of being resistant to empirical therapy for managing acute cholecystitis if they had a higher Charlson comorbidity index, previous ERCP and longer preoperative antibiotic therapy, and thus intraoperative cultures should be considered for guided antibiotic therapy. Trial registration number: NCT06314399.

## 1. Introduction

Laparoscopic cholecystectomy is one of the most frequent surgical procedures performed worldwide as treatment for patients suffering from benign biliary pathology. In most of these cases, bile fluid is aseptic, however, in a varied range of up to 4–70% of cases, bacterial colonization of bile can occur [1,2].

Some strategies have been created in an effort to characterize most frequent microorganisms and antimicrobial resistance patterns to better direct rational antibiotic therapy selection and reduce infectious and postoperative complications. Present studies that document positive bile cultures have documented *Enterobacteriaceae* such as *Escherichia coli*, *Klebsiella* spp. and *Enterobacter* spp., as the most frequently responsible microorganisms in accordance with the biliary tree relation to the gastrointestinal tract. However, this may vary considering population, geographic location, time, individual patient history and institution, and recent trends in resistant bacteria strains [3,4,5].

The 2018 current Tokyo guidelines for the management of acute cholecystitis suggest empirical treatment based on severity of the disease and most commonly found microorganisms (*Enterobacteriaceae* specifically *Escherichia* and *Klebsiella*) with penicillin, cephalosporin, fluoroquinolone, monobactam and carbapenem as valid strategies [6,7]; however, current trends in global bacterial resistance such as extended spectrum beta lactamase production (ESBL) and vancomycin resistant enterococci reflect the need to better characterize bile microbiology based on individual patient characteristics, geographic factors, previous exposure to antibiotics and type of biliary disease in which empirical therapy may not correspond as appropriate management in patients with risk factors for resistant microorganisms.

Bile culture during cholecystectomy becomes, then, a useful diagnostic tool hoping to better address these needs, obtaining an individualized sample of patient microbiology; however, it is not routinely utilized except in cases of perforation, necrosis and emphysematous changes, and there are no clear indications on patients that may require this additional characterization. As such, this study aims to determine bile microbiology and describe factors associated with antimicrobial resistance in positive bile cultures from patients taken to laparoscopic cholecystectomy.

## 2. Materials and Methods

### 2.1. Study Design and Patients

A single-center, observational, analytical prospective cohort study was designed. Between April 2024 and October 2024, 703 bile cultures were taken from consecutive patients taken to laparoscopic cholecystectomy that met eligibility criteria [8,9]. Among these, 226 bile cultures were positive for at least one microorganism. Data for all variables were collected in an anonymous database using REDCap version 16.0.12 electronic data capture tools [10]. This study was reviewed and approved by the Ethics Committee of Universidad del Rosario (approval number 2555-CV1837), approved on 21 February 2024. We followed the STROCSS (Strengthening the reporting of cohort studies in surgery) guideline to report this study [11].

### 2.2. Inclusion and Exclusion Criteria

All patients included in the study were adults over 18 years of age who underwent laparoscopic cholecystectomy at any institution within our Hospital Network. Eligibility criteria required that patients had signed written informed consent and had both bile culture and antimicrobial susceptibility testing (antibiogram) results available. Patients were excluded if they underwent laparoscopic cholecystectomy combined with another concomitant surgical procedure—such as gastrectomy, pancreatoduodenectomy, oesophagectomy, splenectomy, abdominal wall reconstruction or colectomy—with the exception of umbilical herniorrhaphy. Additional exclusion criteria included the absence of a postoperative follow-up appointment or a previously documented diagnosis of gallbladder or biliary tract malignancy.

### 2.3. Bile Culture and Antibiotic Susceptibility Testing

Bile cultures were taken intraoperatively from the surgical specimen, labelled anonymously and sent for analyses in our institutional laboratory. The bile sample was obtained by puncturing the gallbladder wall with a #14 French catheter through the skin. After obtaining the sample, the puncture orifice was occluded with grasper forceps to avoid uncontrolled bile spillage. In cases of bile spillage, the abdominal cavity was adequately cleaned. The bile samples were inoculated on agar plates using MacConkey, thioglycolate and both microaerophilic and GasPak anaerobic chocolate agar plates. They were then incubated for 24, 48 and 72 h in an aerobic environment and for up to 240 h in an anaerobic environment. For positive cultures, the antibiogram was processed through the BD Phoenix M50 Automated Microbiology System from Beckton Dickinson (Loveton Cir, MD, 2115, USA), and carbapenemic resistance was detected through polymerase chain reaction. If any yeast growth was detected, the agar was sent to a reference laboratory, where it was processed through a microdilution technique [8,9].

A culture was defined as “resistant” if any of the microorganisms that grew in it was not susceptible to empiric antibiotic therapy according to the 2018 Tokyo Guidelines for the management of acute cholecystitis. As the wide spectrum of available therapies were available, in order to provide clinical relevance in our institution’s setting we considered a culture as “resistant” if they were resistant to ampicillin/sulbactam for cases of mild acute cholecystitis and piperacillin/tazobactam for cases of moderate and severe cholecystitis, as these are the first-line agents provided to patients in our setting.

### 2.4. Statistical Analysis

Qualitative variables were represented by frequencies and percentages when evaluating demographic, clinical and surgical outcomes and for bile culture characterization. For quantitative variables, central tendency and dispersion measures were presented depending on their distribution and nature (mean and standard deviation, or median and interquartile ranges). Variable normalization was assessed using the Shapiro-Wilk test. A univariate analysis was performed with a χ^2^ test for categorical variables and with the Mann–Whitney test for continuous variables to compare differences between groups based on if the bile culture was susceptible or resistant to empiric antibiotic therapy, as the variables did not meet the assumption of normality. A binomial logistic regression model was constructed using a stepwise selection method to explore the factors associated with the presence of a resistant pattern in bile culture.

A *p*-value below 0.05 was deemed as statistically significant.

All analyses were carried out using R studio version 2023.12.1+402.

## 3. Results

**Figure 1:**
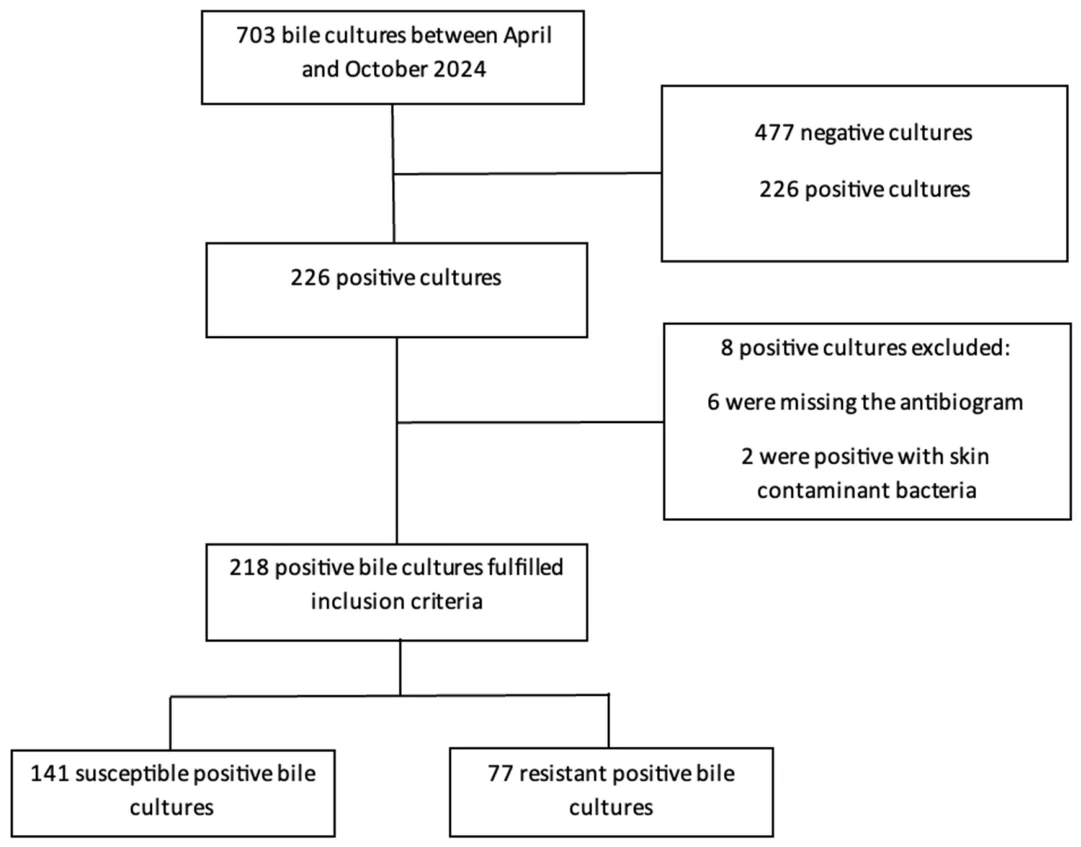
Study selection process flowchart.

A total of 218 positive bile cultures were included in the study, with a flowchart showcasing the selection process (Figure 1). The median age was 67.50 (IQR: 57.00–77.00) years, and the subjects were predominantly female (65.2%). Table 1 describes other demographic, clinical and surgical characteristics divided depending on if the bile culture was susceptible or resistant.

The incidence of resistant cultures was 35.32%. Patients’ demographic and clinical characteristics, sex, body mass index, preoperative inflammatory response markers, pericholecystic collection, previous history of cholecystitis, antibiotic use in the past days, Tokyo severity of cholecystitis, gallbladder wall thickness, cholecystostomy and intraoperative cholecystectomy difficulty findings were not different between groups. Findings of gallbladder perforation were deemed more frequent in the susceptible group but not statistically significant. In the resistant group, older age (*p* = 0.001), higher ASA classification (*p* = 0.005) and higher Charlson comorbidity index (*p* ≤ 0.001), higher bilirubin (*p* = 0.009) and alkaline phosphatase (*p* = 0.001), findings of wider bile duct diameter (*p* ≤ 0.001), preoperative endoscopic retrograde cholangiopancreatography (ERCP) (*p* ≤ 0.001), preoperative duration of antibiotic therapy (*p* ≤ 0.001) and time from admission to procedure (*p* ≤ 0.001) were more frequent and statistically significant compared to the susceptible group.

When analyzing postoperative outcomes between both groups, no statistically significant differences were found between type of cholecystectomy or approach, major complications, drain use, number of microorganisms tested as positive in the cultures, infectious complications and mortality (Table 2).

**Figure 2:**
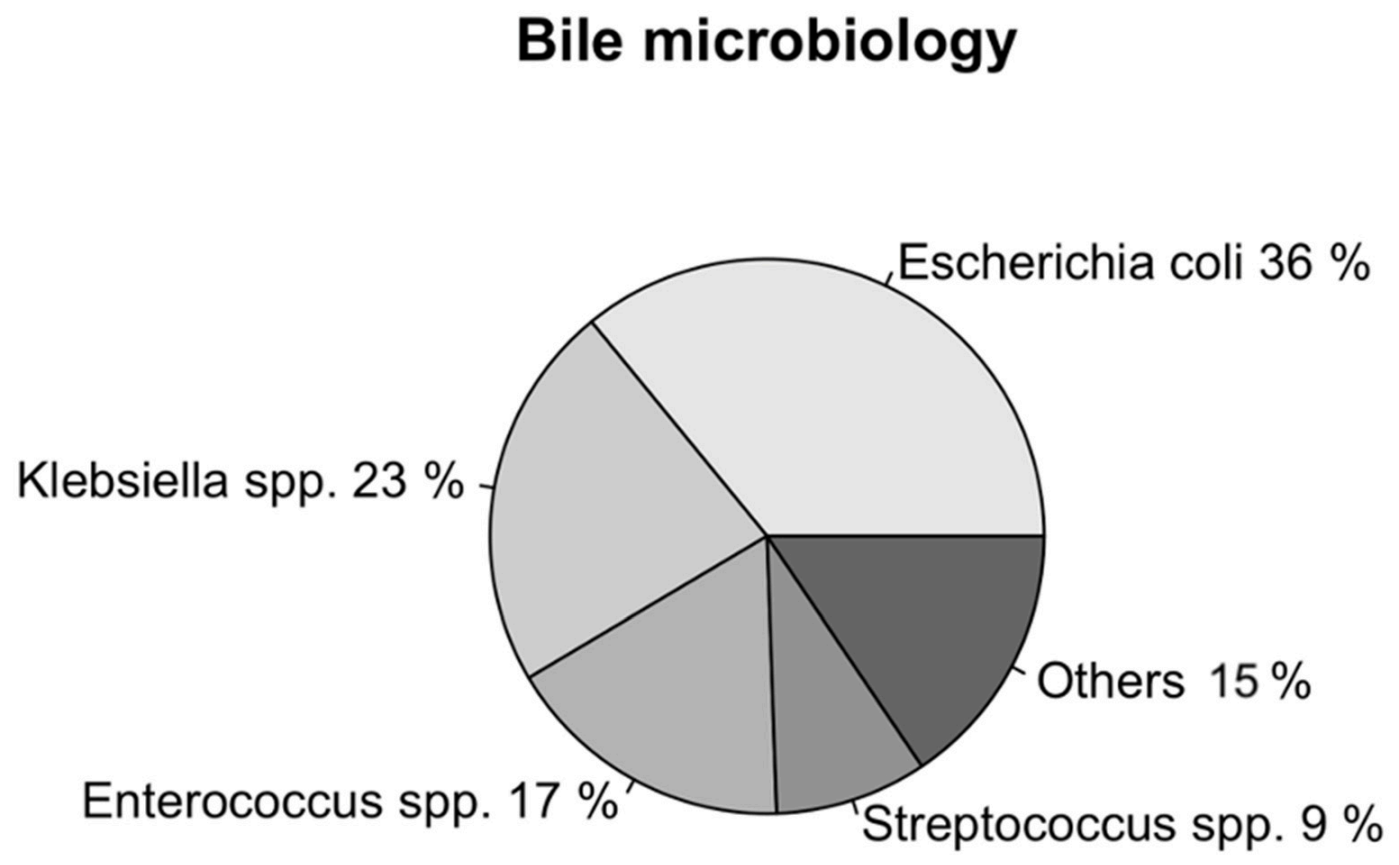
Bacteria genus frequency in positive bile cultures.

Regarding bile culture microbiology, in total 270 microorganisms were found in the 218 cultures, considering some were polymicrobial. Most microorganisms were gram-negative *Enterobacteriaceae*, with the most frequent microorganism being *Escherichia coli* (36%), followed by *Klebsiella pneumoniae* (16.7%), *Enterococcus faecium* (7.4%) and *Enterococcus faecalis* (5.2%). Gram-positive cocci such as streptococcus were present to a lesser extent. A graphic showcasing the most frequent genus of bacteria can be seen in Figure 2. Further information on specific microorganisms, number of microorganisms found in cultures and their resistant patterns can be found as Supplementary Material.

**Figure 3:**
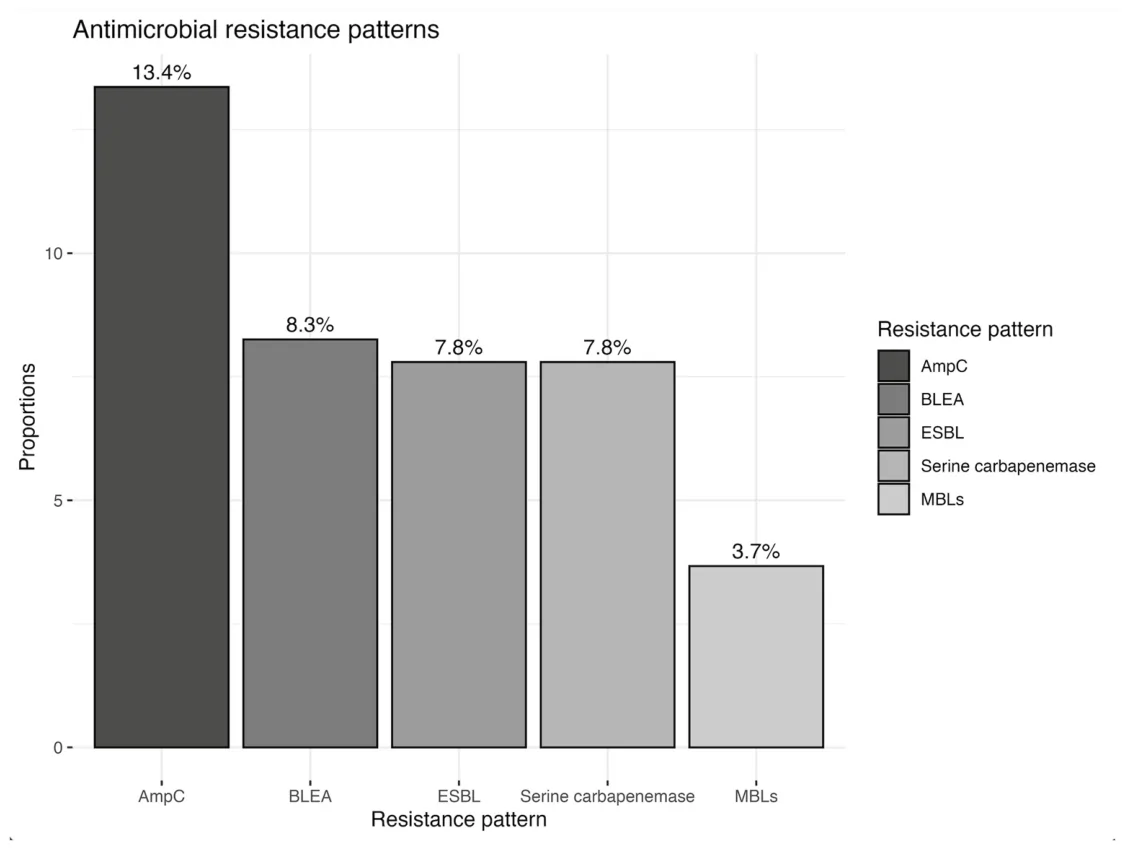
Most commonly found resistance patterns in positive bile cultures.

When testing for antimicrobial resistance, most frequently found resistance patterns were those associated with gram-negative bacteria, which is concordant with bile culture microbiology. The AmpC resistance pattern was most commonly found, representing 13.4% of typified bacteria, followed by broad-spectrum beta-lactamases (BSBL) 8.3%, extended spectrum beta lactamases (ESBL) 7.8% and carbapenemase producing bacteria 7.8%, both serine and metallocarbapenemases (MBLs) (Figure 3). Some gram-positive resistance strains such as methicillin-resistant staphylococcus aureus (MRSA), vancomycin resistant (VMR) and macrolide-lincosamide-streptrogramin-B (MLSB) were found to a much lesser extent. BSLB were considered as microorganisms that were resistant to aminopenicillins and first-generation cephalosporins.

**Figure 4:**
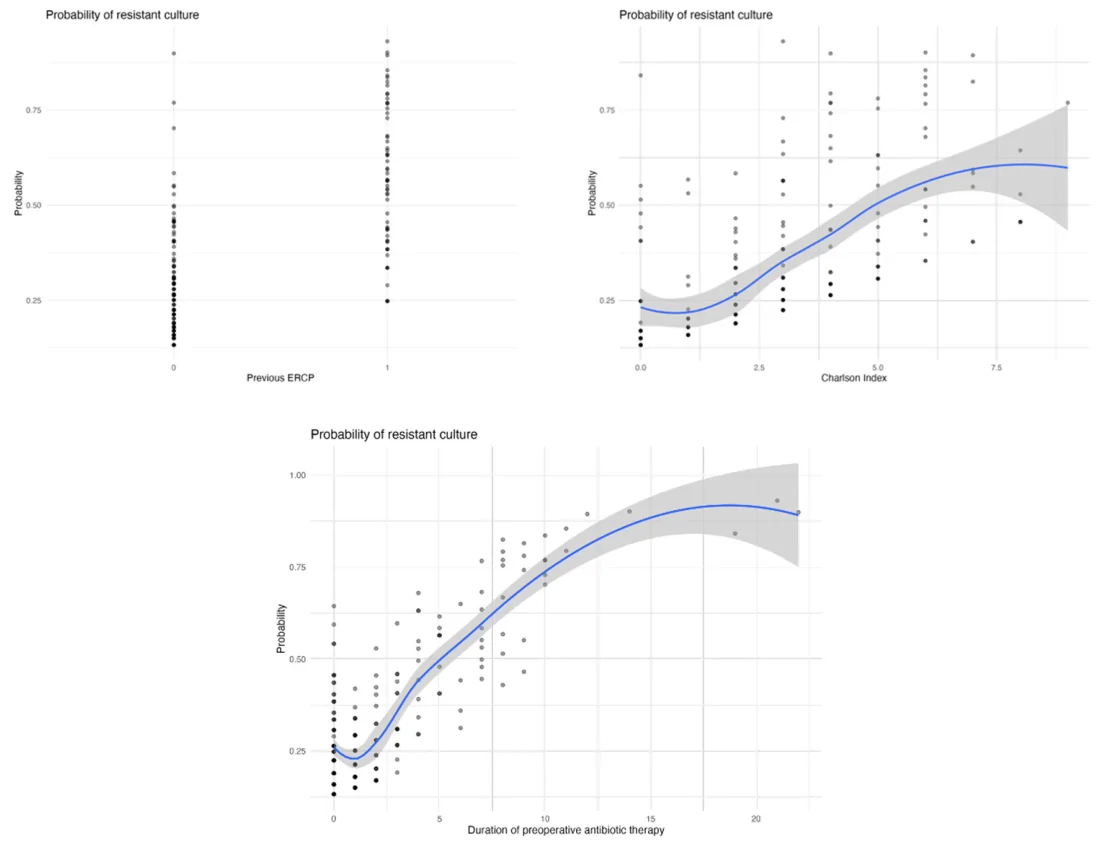
Scatter plot for previous ERCP and sigmoid curves for Charlson comorbidity index and duration of preoperative antibiotic therapy.

**Figure 5:**
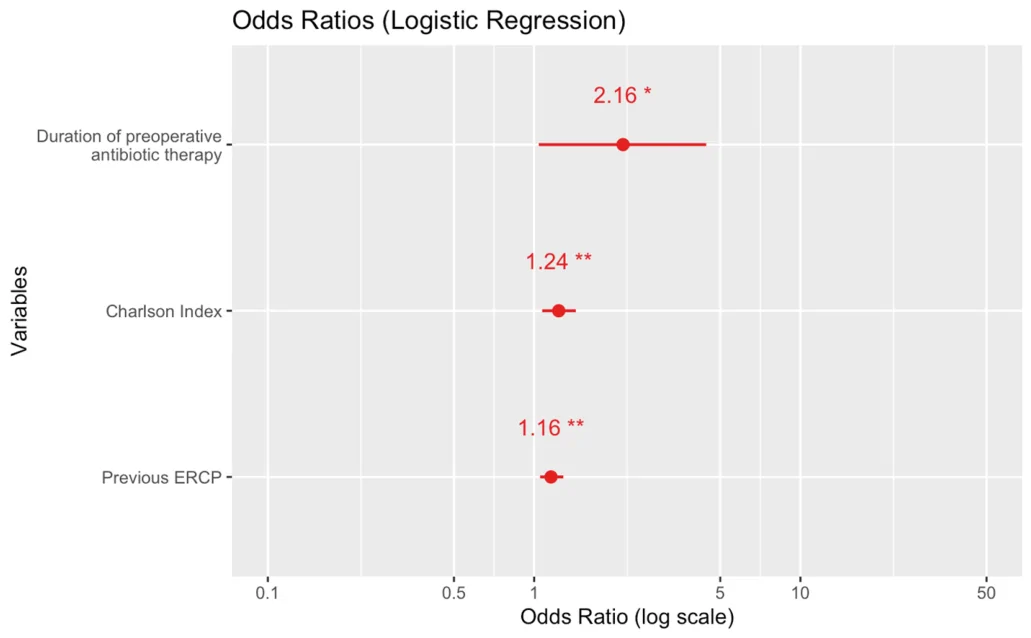
Odds Ratio plot for factors associated with presenting a resistant culture.

A binomial logistic regression analysis was performed to explore risk factors associated with having a “resistant” bile culture. We found that variables independently associated with a resistant culture were previous ERCP (OR 2.16, 95% CI 1.04–4.43, *p* = 0.037), a higher Charlson Comorbidity Index (OR 1.24, 95% CI 1.07–1.43, *p* = 0.004) and longer duration of preoperative antibiotic therapy (OR 1.16, 95% CI 1.05–1.29, *p* = 0.004). Figure 4 illustrates the distribution of these variables according to the presence or absence of resistance. Figure 5 includes the Odds Ratio plot for all three variables.

## 4. Discussion

This study had an incidence of resistant bile cultures of 35.32%, which is consistent with other studies on bile microbiology, reporting ranges of colonization between 4 and up to 70% of patients [1,2]. Our most frequent microorganisms were gram-negative *Enterobacteriaceae Escherichia coli*, *Klebsiella* spp. and *Enterococcus* spp., presenting the following resistance patterns: AmpC (13.4%), BSBL (8.3%), ESBL (7.8%) and carbapenemase producing bacteria (7.8%), both serine and metallocarbapenemases (MBLs).

This microorganism trend is in accordance with previous studies seeking to describe bile microbiology in patients with acute cholecystitis as described in the 2018 Tokyo Guidelines [7]. We observed a higher prevalence of Enterococcus in our cohort. Notably, a 12-year longitudinal study by Kwon et al. also documented a progressive increase in Enterococcus isolates over time, along with a rising proportion of vancomycin-resistant strains [4].

What has been found to be varied among studies is the prevalence of different resistance patterns among regions, especially ESBL-producing bacteria. A study by Farhana, et al. documented a frequency of ESBL of 10.96% and carbapenemase producers of 16.44% in cholecystectomized patients, which is significantly higher compared to our documented frequencies [12]. In a German study, 31.2% of *E. coli* were ESBL-producing, with 70.0% in Korean university medical center [4,6] and 66% in Indian medical college hospital [7]. And while typical strains are mostly sensitive to carbapenem treatment and piperacillin/tazobactam, with low susceptibility to ampicillin, quinolones and ceftriaxone considering currently described trends, around 20% of our cultures were reported as resistant to piperacillin/tazobactam regardless of a lower resistant strain prevalence, without encountering any association with severity of disease and previous antibiotic use. This may indicate the need for a more careful selection of empirical therapy and/or recommending guided management by an infectious disease specialist by performing periodic antibiotic susceptibility testing during laparoscopic cholecystectomy in this group of patients [12,13,14,15].

Factors such as age, comorbidities, previous endoscopic procedure and bile duct instrumentation have already been reported by various authors and are associated with a higher risk of presenting bacterial culture colonization [16,17]. In our study, risk factors independently associated with presenting a resistant culture were previous ERCP, higher Charlson comorbidity index and prolonged preoperative exposure to antibiotic therapy. However, evidence on specific antibiotic resistant trend patterns is scarce [15,16,17,18]. This considered, risk factors for presenting bactibilia are clear, but those related to resistant patterns are not clearly defined but may be similar.

A 2024 study by Miutescu, et al., studying patients with acute cholangitis found that having been taken to laparoscopic cholecystectomy posed a higher risk of presenting multidrug resistance patterns in bile cultures, which extrapolated to our study can correspond to having previous ERCP as a risk factor for resistance [14]. Age has also been described as a risk factor for presenting resistant strains, which was concordant with the one documented in our study [15,19,20]. The burden of comorbidities in presenting antibiotic-resistant organisms has already been studied and is mainly attributed to the patient’s higher exposure to healthcare environments, which generates a higher predisposition to colonization by more resistant pathogens [21]. This relationship has already been studied amongst patients that have higher morbidity burdens, such as the institutionalized elderly with urinary tract infections, cirrhosis and pneumoniae, amongst others [21,22,23]. A recent study also considered the change in biliary flora in patients treated with oral fluoroquinolones owing to changes in resistant bile microbiology for pneumoniae, urinary tract and soft tissue infections [24].

Another worrisome aspect we considered in our study were current growing trends in multidrug resistance patterns and infections by opportunistic microorganisms derived from inappropriate use of antibiotics [25]. This is considering some surgeons utilize preoperative prophylactic antibiotics in severe cases of acute cholecystitis and extend postoperative antibiotic therapy if evolution is unfavorable or if bile spillage happens during surgery hoping to reduce infectious complications and improve patient condition despite the lack of robust evidence suggesting an additional benefit [26,27]. Nonetheless, recent studies favor shorter courses of antibiotics due to their non-inferiority to longer courses [28,29]. It has been proven that patients that are more exposed to longer antibiotic regimens have an increased risk of developing resistant strains due to constant therapeutic exposure [30]. Nonetheless, recent studies favor shorter courses of antibiotics due to their non-inferiority to longer courses [28,29]. This association between prolonged antibiotic exposure was evidenced in our study in patients that had a higher time from admission to the procedure (and thus probably received prolonged preoperative antibiotic therapy) and the duration of antibiotic therapy, both variables being linked with the appearance of a resistant culture, without differences in surgical complications and hospital stay between both groups. The routine use of prolonged preoperative antibiotics does not reduce adverse outcomes while it may favor the appearance of resistant pathogens and presenting multidrug resistant infectious diseases in the future.

This study has some limitations. First, it was performed in a single center with a small cohort, indicating the possibility of a selection bias and its limitation to our geographical and population demographic as they are based on bile cultures performed at our institution. Multicenter and multinational studies should be performed to better represent general bile microbiological patterns. Second, cultures were based on being resistant considering empirical therapy administered in our center, which may exclude valuable results on the efficacy of other recommended empirical therapies documented in current literature and may not be externally validated in other institutions with different treatment regimens. Additionally, the indication for preoperative ERCP, including the presence or absence of cholangitis, was not systematically recorded. Therefore, ERCP may represent a surrogate marker of underlying biliary disease severity and infection. In addition, ERCP-related biliary instrumentation may promote bacterial colonization through duodenobiliary reflux. Consequently, we were unable to determine whether the observed association reflects the effect of the procedure itself, underlying cholangitis, or a combination of both factors. Nonetheless, we consider these results valuable to better document the behavior of bile colonization in patients with benign biliary disease to improve the selection of a wider-spectrum empirical therapy in patients with risk factors for resistant cultures.

The presence of risk factors—such as prior ERCP, a higher Charlson comorbidity index, and prolonged preoperative antibiotic exposure—should alert clinicians to the possibility of resistant organisms. In these patients, a broader-spectrum empiric antibiotic regimen may be warranted to reduce the risk of ineffective therapy and improve antimicrobial management. On the other hand, we suggest a more selective and restrictive use of preoperative antibiotics in patients with symptomatic cholelithiasis or mild to moderate acute cholecystitis (without complications), given that clinically significant differences in outcomes may not be expected.

## 5. Conclusions

Bile microorganisms found consisted mostly of *Enterobacteriaceae*, which is consistent with previous findings in the current literature and their origin in the gastrointestinal tract. Most commonly found resistance patterns include AmpC, ESBL and carbapenemase-producing pathogens. Patients have a higher probability of presenting antimicrobial patterns resistant to empirical therapy for acute cholecystitis if they had a higher Charlson comorbidity index score, were taken to preoperative ERCP and if they were exposed to prolonged preoperative empiric antibiotic therapy.

## Figures and Tables

**Table 1:** Demographic, clinical, paraclinical and intraoperative characteristics based on whether the bile culture was resistant or susceptible to empiric antibiotic therapy.

	N (%)	Susceptible Culture (n = 141)	Resistant Cultures (n = 77)	*p* Value
Age (median)(IQR)(years)	67.50 (57.00–77.00)	63.00 (54.00–74.00)	72.00 (61.00–80.00)	0.001 *
Sex				1.000
Female	142 (65.2)	92 (65.2)	50 (64.9)	
Male	76 (34.8)	49 (34.8)	27 (35.1)	
Body mass index (median)(IQR)(kg/m^2^)	26.20 (23.58–29.76)	26.67 (23.88–30.67)	25.24 (23.39–28.34)	0.066 *
ASA				**0.005**
I	62 (28.5)	46 (32.6)	16 (20.8)	
II	90 (41.3)	61 (43.3)	29 (37.7)	
III	56 (25.7)	32 (22.7)	24 (31.2)	
IV	10 (4.5)	2 (1.4)	8 (10.3)	
Co-morbidity				
Arterial hypertension	106 (48.7)	59 (41.8)	47 (61)	**0.010**
Diabetes mellitus	49 (22.5)	27 (19.1)	22 (28.6)	0.155
Chronic obstructive pulmonary disease	28 (12.8)	11 (7.8)	17 (22.1)	0.005
Chronic kidney disease	8 (3.7)	4 (2.8)	4 (5.2)	0.611
Cardiovascular disease	32 (14.7)	13 (9.2%)	19 (24.7)	0.004
Charlson comorbidity index (media)(IQR)(points)	3.00 (1.00–4.00)	2.00 (1.00–4.00)	4.00 (2.00–5.00)	<0.001 *
Pre-operative laboratories (median)(IQR)				
Leukocytes (× 10^3^)	10.19 (7.20–13.41)	10.23 (7.62–13.87)	9.69 (6.87–13-04)	0.197 *
Hemoglobin (mg/dL)	14.70 (13.60–15.80)	14.80 (13.80–15.80)	14.30 (13.00–15.90)	0.230 *
Bilirubin (mg/dL)	1.25 (0.70–2.28)	1.08 (0.68–1.80)	1.56 (0.78–3.22)	0.009 *
Alkaline phosphatase (mg/dL)	129.0 (86.50–204.0)	113.0 (83.07–181.25)	163.70 (101.00–267.00)	0.001 *
C-reactive protein (mg/dL)	3.91 (1.12–13.55)	3.92 (0.66–9.34)	3.91 (1.91–14.55)	0.269 *
Imaging findings				
Bile duct diameter (median)(IQR)(mm)	4.20 (4.00–6.00)	4.00 (4.00–6.00)	5.00 (4.00–8.00)	0.001 *
Gallbladder wall thickness (median)(IQR)(mm)	4.00 (3.00–5.00)	4.00 (2.00–5.00)	4.00 (3.00–5.00)	0.097 *
Pericholecystic collection	17 (7.8)	11 (7.8)	6 (7.8)	0.998
Perforation	9 (4.1)	9 (6.4)	0 (0.0)	0.056
History of previous episodes of cholecystitis				0.416
No	196 (89.9)	129 (91.5)	67 (87.0)	
Yes	22 (10.1)	12 (8.5)	10 (13.0)	
Antibiotic use in the past 90 days				0.948
No	169 (77.5)	110 (78.0)	59 (76.6)	
Yes	49 (22.5)	31 (22.0)	18 (23.4)	
Tokyo severity				0.894
Without cholecystitis	102 (46.8)	67 (47.5)	35 (45.5)	
I	34 (15.6)	22 (15.6)	12 (15.6)	
II	45 (20.6)	27 (19.2)	18 (23.4)	
III	37 (17)	25 (17.7)	12 (15.6)	
Pre-operative ERCP				**<0.001**
No	157 (72)	115 (81.6)	42 (54.5)	
Yes	61 (28)	26 (18.4)	35 (45.5)	
Cholecystostomy				0.251
No	214 (98.2)	140 (99.3)	74 (96.1)	
Yes	4 (1.8)	1 (0.7)	3 (3.9)	
Preoperative antibiotic				**0.025**
No	82 (37.1)	62 (43.6)	20 (26)	
Yes	135 (62.9)	78 (55.7)	57 (74)	
Duration of preoperative antibiotic (median) (IQR) (days)	1.00 (0.00–4.00)	1.00 (0.00–2.00)	3.00 (0.00–7.00)	**<0.001**
Intraoperative findings according to Nassar score				0.492
1	38 (17.4)	22 (15.6)	16 (20.8)	
2	65 (29.8)	39 (27.7)	26 (33.8)	
3	40 (18.4)	27 (19.1)	13 (16.9)	
4	55 (25.2)	23 (16.3)	12 (15.6)	
5	40 (18.4)	30 (21.3)	10 (13.0)	
Time from admission to procedure (median) (IQR) (days)	4.00 (2.00–7.00)	3.00 (2.00–5.00)	5.00 (3.00–9.00)	<0.001 *

*p* values were obtained using the chi-squared test. * *p* values were obtained using the Mann–Whitney test. Bold values indicate statistically significant *p* values (*p* < 0.05).

**Table 2:** Surgical outcomes based on whether the bile culture was resistant or susceptible to empiric antibiotic therapy.

	N (%)	Susceptible Culture (n = 141)	Resistant Cultures (n = 77)	*p* Value
Conversion to open				1.000
No	214 (98.2)	138 (97.9)	76 (98.7)	
Yes	4 (1.8)	3 (2.1)	1 (1.3)	
Type of cholecystectomy				0.934
Total	202 (92.6)	130 (92.2)	72 (93.5)	
Subtotal	16 (7.4)	11 (7.8)	5 (6.5)	
Surgical site infection				1.000
No	213 (97.7)	138 (97.9)	75 (97.4)	
Yes	5 (2.3)	3 (2.1)	2 (2.6)	
Drain use				0.357
No	197 (90.3)	125 (88.7)	72 (93.5)	
Yes	21 (9.7)	16 (11.3)	5 (6.5)	
Number of positive microorganisms in culture				0.375
One	171 (78.4)	114 (80.9)	57 (74.0)	
Two	43 (19.7)	24 (17.0)	19 (24.7)	
Three	4 (1.9)	3 (2.1)	1 (1.3)	
Surgical time (median)(IQR)(minutes)	80.00 (57.25–105.75)	78.00 (60.00–111.00)	85.00 (55.00–101.00)	0.503 *
Postoperative hospital stay (median)(IQR)(days)	1.00 (0.00–3.00)		1.00 (0.00–3.00)	0.847 *
Major complication (Clavien-Dindo ≥3)				0.797
No	204 (93.6)	131 (92.9)	73 (94.8)	
Yes	14 (6.4)	10 (7.1)	4 (5.2)	
30-day mortality				1.000
No	214 (98.2)	138 (97.9)	76 (98.7)	
Yes	4 (1.8)	3 (2.1)	1 (1.3)	

*p* values were obtained using the Chi-squared test. * *p* values were obtained using the Mann–Whitney test.

## Data Availability

Demographic, clinical, paraclinical, intraoperative and surgical outcome data used in this study are available online (Dataverse), along with the code for logistic regression and internal validation. https://doi.org/10.34848/PYATTI (accessed on 25 May 2026). This study was registered on ClinicalTrials.gov in March 2024 under the registration number NCT06314399. The study protocol has been published and is available at the following DOI: 10.1136/bmjopen-2024-086655 (accessed on 25 May 2026).

## Supplementary Materials

The following supporting information can be downloaded at: https://www.mdpi.com/article/10.3390/antibiotics15080717/s1, Table S1: List of cultures and identified resistance patterns.

## Abbreviations

The following abbreviations are used in this manuscript:

ESBL	Extended spectrum beta lactamase
ASA	American Society of Anesthesiologists
ERCP	Endoscopic retrograde cholangiopancreatography
BSBL	Broad-spectrum betalactamase
MBL	Metallocarbapenemases
MRSA	Methicillin-resistant staphylococcus aureus
VMR	Vancomycin resistant
MLSB	Macrolide-lincosamide-streptogramin B
