# Supplementary material for: Bile Microbiology and Risk Factors Associated with Antibiotic Resistance Patterns in Patients Taken to Laparoscopic Cholecystectomy: A Prospective Cohort Study
Source: Antibiotics (Basel). 2026 Jul 24;15(8):717. doi: 10.3390/antibiotics15080717 (PMC13509471; doi:10.3390/antibiotics15080717)
Supplement: Supplementary file 1 [file antibiotics-15-00717-s001.zip › antibiotics-4301717-supplementary.pdf]

Culture number	Number of microorganisms	Microorganism 1	Microorganism 2	Microorganism 3	Resistant pattern	KPC_B	KPC_AC	ESBL	BSBL	AmpC	MRSA	VMR	MLSB	Others	Preoperative antibiotic given?	Days of antibiotic
1	1	streptococcus anginosus	No	No	No	No	No	No	No	No	No	No	No	No	No	0
2	2	escherichia coli	enterococcus faecium	No	Yes	Yes	No	No	No	No	No	Yes	No	No	Yes	2
3	1	citrobacter koseri	No	No	Yes	No	No	No	No	Yes	No	No	No	No	Yes	1
4	1	haemophilus parainfluenzae	No	No	No	No	No	No	No	No	No	No	No	No	Yes	1
5	1	escherichia coli	No	No	No	No	No	No	No	No	No	No	No	Ciprofloxacin resistant	No	0
6	1	escherichia coli	No	No	No	No	No	No	No	No	No	No	No	No	Yes	2
7	1	kluyvera ascorbata	No	No	No	No	No	No	No	No	No	No	No	Non applicable	No	0
8	1	escherichia coli	No	No	No	No	No	No	No	No	No	No	No	No	Yes	4
9	2	escherichia coli	enterococcus cecorum	No	No	No	No	No	No	No	No	No	No	Resistant to ciprofloxacin	Yes	4
10	1	enterococcus faecium	No	No	No	No	No	No	No	No	No	No	No	No	Yes	2
11	1	kluyvera ascorbata	No	No	No	No	No	No	No	No	No	No	No	IRT	Yes	21
12	2	klebsiella oxytoca	escherichia coli	No	No	No	No	No	No	No	No	No	No	No	No	0
13	1	enterococcus faecium	No	No	No	No	No	No	No	No	No	No	No	No	No	0
14	1	escherichia coli	No	No	Yes	No	No	No	No	Yes	No	No	No	No	No	0
15	1	klebsiella oxytoca	No	No	No	No	No	No	No	No	No	No	No	No	No	1
16	1	enterococcus faecium	No	No	No	No	No	No	No	No	No	No	No	No	No	0
17	1	enterococcus faecium	No	No	No	No	No	No	No	No	No	No	No	No	Yes	2
18	1	klebsiella pneumoniae	No	No	Yes	Yes	Yes	No	No	No	No	No	No	No	Yes	8
19	1	streptococcus parasanguinis	No	No	No	No	No	No	No	No	No	No	No	No	No	0
20	1	klebsiella pneumoniae	No	No	Yes	No	No	Yes	No	No	No	No	No	No	Yes	14
21	1	escherichia coli	No	No	No	No	No	No	No	No	No	No	No	Simple penicillinases producer	Yes	5
22	2	enterococcus faecalis	klebsiella pneumoniae	No	No	No	No	No	No	No	No	No	No	No	No	0
23	1	enterococcus hirae	No	No	No	No	No	No	No	No	No	No	No	No	No	0
24	1	escherichia coli	No	No	No	No	No	No	No	No	No	No	No	No	Yes	3
25	2	enterobacter kobei	enterococcus casseliflavus	No	No	No	No	No	No	Yes	No	Yes	No	Resistant to linezolid	Yes	1
26	1	enterococcus faecium	No	No	No	No	No	No	No	No	No	Yes	No	No	No	0
27	1	klebsiella pneumoniae	No	No	Yes	No	Yes	No	No	No	No	No	No	No	Yes	8
28	2	klebsiella pneumoniae	enterococcus faecium	No	Yes	Yes	Yes	No	No	No	No	No	No	Resistant to high-level streptomycin	No	0
29	1	klebsiella pneumoniae	No	No	No	No	No	No	No	No	No	No	No	No	Yes	5
30	1	klebsiella pneumoniae	No	No	Yes	No	Yes	No	No	No	No	No	No	No	Yes	10
31	2	serratia marcescens	enterococcus faecium	No	Yes	No	No	No	No	Yes	No	No	Yes	Resistant to high-level streptomycin and gentamicin	Yes	1
32	1	escherichia coli	No	No	Yes	No	No	No	No	Yes	No	No	No	No	Yes	6
33	1	streptococcus sanguinis	No	No	No	No	No	No	No	No	No	No	No	No	No	0
34	1	escherichia coli	No	No	No	No	No	No	No	No	No	No	No	No	Yes	1
35	1	escherichia coli	No	No	No	No	No	No	No	No	No	No	No	No	Yes	1
36	1	klebsiella pneumoniae	No	No	No	No	No	No	No	No	No	No	No	No	No	0
37	2	morganella morganii	streptococcus gallolyticus	enterococcus gallinarum	Yes	No	No	No	No	Yes	No	No	Yes	No	No	0
38	1	escherichia coli	No	No	Yes	No	No	No	Yes	No	No	No	No	No	Yes	5
39	2	escherichia coli	klebsiella oxytoca	No	Yes	No	No	Yes	No	No	No	No	No	No	Yes	4
40	1	klebsiella pneumoniae	No	No	Yes	Yes	Yes	No	No	No	No	No	No	No	Yes	22
41	1	escherichia coli	No	No	No	No	No	No	Yes	No	No	No	No	No	Yes	12
42	1	escherichia coli	No	No	No	No	No	No	No	No	No	No	No	No	No	0
43	1	streptococcus sanguinis	No	No	No	No	No	No	No	No	No	No	No	Penicillases producer	Yes	2
44	1	pseudomonas fluorescens	No	No	No	No	No	No	No	No	No	No	No	Resistant to aztreonam	No	0
45	2	klebsiella pneumoniae	enterococcus gallinarum	No	Yes	No	No	No	No	No	No	Yes	No	Simple penicillinases producer (enterococcus)	No	0
46	1	escherichia coli	No	No	Yes	No	No	No	Yes	No	No	No	No	No	Yes	7
47	1	escherichia coli	No	No	No	No	No	No	No	No	No	No	No	No	Yes	1
48	2	escherichia coli	klebsiella pneumoniae	No	No	No	No	No	No	No	No	No	No	No	No	0
49	1	serratia marcescens	No	No	Yes	No	No	No	No	Yes	No	No	No	No	Yes	6
50	1	klebsiella variicola	No	No	No	No	No	No	No	No	No	No	No	No	Yes	1
51	1	klebsiella pneumoniae	No	No	Yes	Yes	Yes	No	No	No	No	No	No	No	Yes	7
52	1	klebsiella oxytoca	No	No	No	No	No	No	No	No	No	No	No	No	Yes	19
53	2	escherichia coli	klebsiella pneumoniae	No	No	No	No	No	No	Yes	No	No	No	AmpC Klebsiella	Yes	2
54	1	streptococcus sanguinis	No	No	No	No	No	No	No	No	No	No	No	No	Yes	1
55	1	escherichia coli	No	No	No	No	No	No	No	Yes	No	No	No	No	No	0
56	1	escherichia coli	No	No	No	No	No	No	No	No	No	No	No	No	Yes	1
57	1	klebsiella pneumoniae	No	No	No	No	No	No	No	No	No	No	No	No	Yes	5
58	2	klebsiella pneumoniae	escherichia coli	No	Yes	No	Yes	No	Yes	No	No	No	No	No	Yes	11
59	1	escherichia coli	No	No	No	No	No	No	No	Yes	No	No	No	No	Yes	9
60	1	raoultella ornithinolytica	No	No	No	No	No	No	No	No	No	No	No	Simple penicillinases producer	Yes	1
61	1	escherichia coli	No	No	No	No	No	No	No	No	No	No	No	Penicillases hyper-producer	No	0
62	2	escherichia coli	klebsiella pneumoniae	No	No	No	No	No	No	No	No	No	No	No	Yes	8
63	1	klebsiella pneumoniae	No	No	Yes	No	Yes	No	No	No	No	No	No	No	Yes	7
64	1	escherichia coli	No	No	Yes	No	No	Yes	No	No	No	No	No	No	No	0
65	1	staphylococcus warneri	No	No	No	No	No	No	No	No	No	No	Yes	No	No	0
66	1	escherichia coli	No	No	No	No	No	No	No	No	No	No	No	No	Yes	10
67	1	escherichia coli	No	No	No	No	No	No	No	No	No	No	No	No	Yes	1
68	1	escherichia coli	No	No	No	No	No	No	No	No	No	No	No	No	Yes	4
69	1	klebsiella pneumoniae	No	No	No	No	No	No	Yes	No	No	No	No	IRT	No	0
70	1	escherichia coli	No	No	Yes	No	No	No	Yes	No	No	No	No	No	Yes	8
71	1	klebsiella variicola	No	No	Yes	No	No	No	Yes	No	No	No	No	No	Yes	6
72	1	escherichia coli	No	No	Yes	No	No	No	No	Yes	No	No	No	No	Yes	3
73	1	enterobacter hormaechei	No	No	No	No	No	No	Yes	No	No	No	No	No	No	0
74	1	streptococcus gallolyticus	No	No	No	No	No	No	No	No	No	No	Yes	No	Yes	1
75	1	enterococcus faecium	No	No	No	No	No	No	No	No	No	No	No	Penicillases hyper-producer	Yes	10
76	1	streptococcus salivarius	No	No	No	No	No	No	No	No	No	No	No	No	No	0
77	2	haemophilus parainfluenzae	enterobacter bugandensis	No	No	No	No	No	No	Yes	No	No	No	No	No	0
78	1	escherichia coli	No	No	No	No	No	No	No	No	No	No	No	No	No	0
79	1	streptococcus salivarius	No	No	No	No	No	No	No	No	No	No	Yes	No	No	0
80	1	enterobacter hormaechei	No	No	Yes	No	No	No	No	Yes	No	No	No	No	Yes	1
81	1	streptococcus mitis oralis	No	No	Yes	No	No	No	No	No	No	No	Yes	Mec gene + MLSB, facultative anaerobic, resistant to betalactamics	Yes	7
82	1	enterococcus faecalis	No	No	No	No	No	No	No	No	No	No	No	No		0
83	1	enterobacter kobei	No	No	Yes	No	No	No	No	Yes	No	No	No	No	Yes	1
84	2	enterococcus faecalis	escherichia coli	No	No	No	No	No	No	No	No	No	No	Simple penicillinases producer	No	0
85	1	escherichia coli	No	No	No	No	No	No	No	No	No	No	No	No	No	0
86	1	enterococcus faecium	No	No	No	No	No	No	No	No	No	No	No	No	No	0
87	1	escherichia coli	No	No	No	No	No	No	No	No	No	No	No	No	Yes	3
88	1	klebsiella pneumoniae	No	No	Yes	Yes	Yes	No	No	No	No	No	No	No	No	0
89	1	escherichia coli	No	No	No	No	No	No	No	No	No	No	No	No	Yes	3
90	1	escherichia coli	No	No	No	No	No	No	No	No	No	No	No	No	Yes	2
91	1	klebsiella oxytoca	No	No	Yes	No	No	No	No	No	No	No	No	Simple penicillinases producer + quinolones	Yes	1
92	1	escherichia coli	No	No	No	No	No	No	No	No	No	No	No	No	Yes	3
93	1	escherichia coli	No	No	Yes	No	No	Yes	No	No	No	No	No	No	Yes	4
94	1	weissella cibaria	No	No	No	No	No	No	No	No	No	No	No	No	No	0
95	1	enterococcus faecalis	No	No	No	No	No	No	No	No	No	No	No	No	Yes	2
96	2	klebsiella pneumoniae	enterococcus gallinarum	No	Yes	No	No	Yes	No	No	No	No	No	VRE	Yes	11
97	1	escherichia coli	No	No	No	No	No	No	No	No	No	No	No	No	No	0
98	1	enterococcus durans	No	No	No	No	No	No	No	No	No	No	No	Resistant to linezolid	Yes	1
99	1	klebsiella oxytoca	No	No	No	No	No	No	No	No	No	No	No	No	Yes	1
100	1	streptococcus sanguinis	No	No	No	No	No	No	No	No	No	No	No	No	Yes	1
101	1	klebsiella pneumoniae	No	No	Yes	No	Yes	No	No	No	No	No	No	No	Yes	9
102	1	escherichia coli	No	No	Yes	No	No	No	Yes	No	No	No	No	No	Yes	2
103	1	aeromonas hydrophila	No	No	Yes	No	No	No	No	Yes	No	No	No	No	Yes	1
104	1	escherichia coli	No	No	Yes	No	No	Yes	No	No	No	No	No	No	Yes	9
105	2	hafnia alvei	streptococcus salivarius	No	Yes	No	No	No	No	Yes	No	No	Yes	No	No	0
106	2	escherichia coli	enterococcus faecalis	No	No	No	No	No	No	No	No	No	No	No	No	0
107	1	escherichia coli	No	No	No	No	No	No	Yes	No	No	No	No	No	Yes	3
108	2	streptococcus anginosus	enterococcus faecalis	No	No	No	No	No	No	No	No	No	No	Intermediate resistance to daptomycin (enterococcus)	Yes	4
109	1	escherichia coli	No	No	No	No	No	No	No	No	No	No	No	Resistant to TMP-SMX	Yes	2
110	1	streptococcus infantarius	No	No	No	No	No	No	No	No	No	No	No	No	No	0
111	2	hafnia alvei	streptococcus anginosus	No	Yes	No	No	No	No	Yes	No	No	No	No	Yes	2
112	1	escherichia coli	No	No	No	No	No	No	No	No	No	No	No	Penicillases hyper-producer	Yes	1
113	1	klebsiella pneumoniae	No	No	Yes	No	No	Yes	No	No	No	No	No	No	Yes	5
114	2	klebsiella variicola	escherichia coli	No	No	No	No	No	No	No	No	No	No	No	No	0
115	1	enterococcus faecalis	No	No	No	No	No	No	No	No	No	No	No	No	No	0
116	1	enterococcus faecium	No	No	No	No	No	No	No	No	No	No	No	No	No	0
117	2	klebsiella pneumoniae	enterobacter hormaenchi	No	Yes	No	No	No	No	Yes	No	No	No	No	Yes	1
118	1	escherichia coli	No	No	No	No	No	No	No	No	No	No	No	Penicillases hyper-producer	.	0
119	1	escherichia coli	No	No	No	Yes	No	Yes	No	No	No	No	No	No	Yes	3
120	1	klebsiella oxytoca	No	No	No	No	No	No	No	No	No	No	No	No	Yes	1
121	1	enterococcus avium	No	No	No	No	No	No	No	No	No	No	No	No	Yes	5
122	1	escherichia coli	No	No	No	No	No	No	No	No	No	No	No	No	Yes	2
123	1	escherichia coli	No	No	No	No	No	No	No	No	No	No	No	No	No	0
124	1	kluyvera intermedia	No	No	No	No	No	No	No	No	No	No	No	Resistant to cefazolin	No	0
125	1	escherichia coli	No	No	No	No	No	No	No	No	No	No	No	No	Yes	3
126	1	escherichia coli	No	No	No	No	No	No	No	No	No	No	No	Resistant to amikacin	No	0
127	1	klebsiella pneumoniae	No	No	Yes	No	No	Yes	No	No	No	No	No	No	Yes	8
128	1	escherichia coli	No	No	No	No	No	No	No	No	No	No	No	No	Yes	1
129	1	kluyvera ascorbata	No	No	Yes	No	No	No	Yes	No	No	No	No	No	Yes	1
130	3	escherichia coli	klebsiella oxytoca	enterococcus faecalis	No	No	No	No	No	No	No	No	No	No	No	0
131	1	klebsiella pneumoniae	No	No	No	No	No	No	No	No	No	No	No	No	Yes	1
132	1	streptococcus mitis/oralis	No	No	Yes	No	No	No	No	No	No	No	Yes	No	Yes	7
133	2	escherichia coli	aeromonas hydrophila	No	Yes	No	No	No	Yes	Yes	No	No	No	No	Yes	1
134	1	klebsiella pneumoniae	No	No	No	No	No	No	No	No	No	No	No	No	No	0
135	1	escherichia coli	No	No	No	No	No	No	No	No	No	No	No	No	No	0
136	2	klebsiella pneumoniae	enterococcus faecalis	No	Yes	No	Yes	No	No	No	No	No	No	No	Yes	7
137	1	escherichia coli	No	No	No	No	No	No	No	No	No	No	No	Resistant to TMP-SMX	Yes	2
138	1	escherichia coli	No	No	No	No	No	No	No	No	No	No	No	No	No	0
139	1	klebsiella pneumoniae	No	No	Yes	No	No	Yes	No	No	No	No	No	No	Yes	7
140	1	escherichia coli	No	No	Yes	No	No	Yes	No	No	No	No	No	No	No	0
141	1	streprococcus sanguinis	No	No	No	No	No	No	No	No	No	No	Yes	No	No	0
142	1	klebsiella pneumoniae	No	No	Yes	No	No	Yes	No	No	No	No	No	No	Yes	5
143	1	escherichia coli	No	No	No	No	No	No	No	No	No	No	No	Penicillases hyper-producer	Yes	1
144	1	morganella morganii	No	No	Yes	No	No	No	No	Yes	No	No	No	No	No	0
145	2	clostridium perfingens	escherichia coli	No	No	No	No	No	No	No	No	No	No	Penicillases hyper-producer	No	0

146	1	escherichia coli	No	No	No	No	No	No	No	No	No	No	No	No	Yes	2
147	1	enterococcus faecium	No	No	No	No	No	No	No	No	No	No	No	No	Yes	3
148	1	escherichia coli	No	No	No	No	No	No	No	No	No	No	No	No	Yes	1
149	2	klebsiella pneumoniae	peptostreptococcus stomatis	No	No	No	No	No	No	No	No	No	No	No	No	0
150	1	klebsiella pneumoniae	No	No	Yes	No	No	Yes	No	No	No	No	No	No	Yes	9
151	2	enterococcus faecium	clostridium perfringens	No	No	No	No	No	No	No	No	No	No	No	No	0
152	1	klebsiella pneumoniae	No	No	Yes	No	Yes	No	No	No	No	No	No	No	Yes	2
153	1	escherichia coli	No	No	No	No	No	No	No	No	No	No	No	No	Yes	1
154	3	citrobacter freundii	klebsiella oxytoca	enterococcus faecium	Yes	No	No	No	No	Yes	No	No	No	Resistant to cefazolin (klebsiella)	Yes	1
155	1	klebsiella oxytoca	No	No	No	No	No	No	No	No	No	No	No	Intermediate resistance to cefazolin	Yes	1
156	1	escherichia coli	No	No	No	No	No	No	No	No	No	No	No	Penicillases hyper-producer	Yes	1
157	2	klebsiella pneumoniae	escherichia coli	No	No	No	No	No	No	No	No	No	No	Resistant to TMP-SMX	No	8
158	1	escherichia coli	No	No	No	No	No	No	No	No	No	No	No	No	Yes	7
159	1	hafnia alvei	No	No	Yes	No	No	No	No	Yes	No	No	No	No	No	0
160	2	klebsiella pneumoniae	escherichia coli	No	No	No	No	No	No	No	No	No	No	Penicillases hyper-producer	Yes	1
161	1	escherichia coli	No	No	No	No	No	No	No	No	No	No	No	Penicillases hyper-producer	Yes	1
162	1	streprococcus parasanguinis	No	No	No	No	No	No	No	No	No	No	No	No	No	0
163	1	escherichia coli	No	No	Yes	No	No	Yes	No	No	No	No	No	No	No	0
164	2	enterococcus faecalis	enterobacter hormaenchi	No	Yes	No	No	No	No	Yes	No	No	No	No	No	0
165	1	klebsiella pneumoniae	No	No	No	No	No	No	No	No	No	No	No	No	Yes	1
166	1	clostridium perfingens	No	No	No	No	No	No	No	No	No	No	No	No	Yes	4
167	1	streptococcus vestibularis	No	No	No	No	No	No	No	No	No	No	No	No	No	0
168	1	haemophilus parainfluenzae	No	No	No	No	No	No	No	No	No	No	No	No	No	0
169	1	escherichia coli	No	No	No	No	No	No	No	No	No	No	No	Penicillases hyper-producer	Yes	2
170	1	klebsiella pneumoniae	No	No	No	No	No	No	No	No	No	No	No	Intermediate resistance to a ampicillin sulbactam	Yes	3
171	1	klebsiella pneumoniae	No	No	Yes	No	Yes	No	No	No	No	No	No	No	Yes	8
172	1	citrobacter braakii	No	No	Yes	No	No	No	No	Yes	No	No	No	No	No	0
173	1	escherichia coli	No	No	No	No	No	No	No	No	No	No	No	Penicillases hyper-producer	Yes	7
174	1	klebsiella pneumoniae	No	No	Yes	No	Yes	No	No	No	No	No	No	No	Yes	10
175	1	enterococcus avium	No	No	No	No	No	No	No	No	No	No	No	Resistant to tetracyclin	No	0
176	1	escherichia coli	No	No	No	No	No	No	No	No	No	No	No	No	No	0
177	1	escherichia coli	No	No	No	No	No	No	No	No	No	No	No	No	Yes	3
178	1	morganella morganii	No	No	Yes	No	No	No	No	Yes	No	No	No	No	No	0
179	1	streprococcus parasanguinis	No	No	No	No	No	No	No	No	No	No	No	No	No	0
180	1	enterococcus faecium	No	No	No	No	No	No	No	No	No	No	No	No	Yes	1
181	1	escherichia coli	No	No	No	No	No	No	No	No	No	No	No	No	No	0
182	1	enterococcus faecium	No	No	No	No	No	No	No	No	No	No	No	No	No	0
183	1	escherichia coli	No	No	No	No	No	No	No	No	No	No	No	No	Yes	4
184	2	escherichia coli	aeromonas veronii	No	Yes	No	No	No	No	Yes	No	No	No	No	No	0
185	1	escherichia coli	No	No	No	No	No	No	No	No	No	No	No	No	Yes	2
186	2	klebsiella pneumoniae	escherichia coli	No	Yes	No	No	Yes	No	No	No	No	No	No	No	0
187	2	klebsiella pneumoniae	enterococcus faecium	No	Yes	No	Yes	No	No	No	No	No	No	Resistant to streptomycin (enterococcus)	Yes	10
188	1	escherichia coli	No	No	Yes	No	No	No	Yes	No	No	No	No	Resistant to ciprofloxacin	Yes	9
189	1	proteus vulgaris	No	No	Yes	No	No	No	No	Yes	No	No	No	No	No	0
190	1	streptococcus gallolyticus	No	No	Yes	No	No	No	No	No	No	No	Yes	No	No	0
191	1	escherichia coli	No	No	No	No	No	No	No	No	No	No	No	Resistant to gentamicin	Yes	4
192	1	escherichia coli	No	No	Yes	No	No	No	No	Yes	No	No	No	No	Yes	2
193	2	leclercia adecarboxylata	streptococcus anginosus	No	No	No	No	No	No	No	No	No	No	Resistant to TMP-SMX	Yes	1
194	1	escherichia coli	No	No	No	No	No	No	No	No	No	No	No	Penicillases hyper-producer	No	0
195	1	escherichia coli	No	No	Yes	No	No	No	No	No	No	No	No	IRT, resistant to TMP-SMX and ciprofloxacin	Yes	2
196	1	enterococcus faecalis	No	No	No	No	No	No	No	No	No	No	No	No	Yes	4
197	2	klebsiella pneumoniae	enterococcus avium	No	Yes	No	No	No	Yes	No	No	No	No	No	No	0
198	1	escherichia coli	No	No	Yes	No	No	Yes	No	No	No	No	No	No	No	0
199	1	morganella morganii	No	No	Yes	No	No	No	No	Yes	No	No	No	No	No	0
200	3	enterococcus faecalis	escherichia coli	enterococcus canintestini	No	No	No	No	No	No	No	No	No	No	No	0
201	1	escherichia coli	No	No	Yes	No	No	No	Yes	No	No	No	No	No	Yes	3
202	3	klebsiella oxytoca	aeromonas caviae	escherichia coli	No	No	No	No	No	No	No	No	No	Penicillases hyper-producer	No	0
203	1	klebsiella pneumoniae	No	No	Yes	No	Yes	No	No	No	No	No	No	No	Yes	1
204	2	escherichia coli	klebsiella pneumoniae	No	No	No	No	No	No	No	No	No	No	No	Yes	3
205	1	kluyvera cryocrescens	No	No	Yes	No	No	No	Yes	No	No	No	No	No	Yes	1
206	1	klebsiella pneumoniae	No	No	No	No	No	No	No	No	No	No	No	No	Yes	3
207	1	streptococcus sanguinis	No	No	No	No	No	No	No	No	No	No	No	No	Yes	2
208	1	enterococcus faecium			No	No	No	No	No	No	No	No	No	No	Yes	6
209	1	escherichia coli	No	No	No	No	No	No	No	No	No	No	No	No	Yes	2
210	2	klebsiella pneumoniae	enterococcus faecalis	No	Yes	Yes	Yes	No	No	No	No	No	No	No	Yes	4
211	1	escherichia coli	No	No	Yes	No	No	No	Yes	No	No	No	No	No	Yes	3
212	2	pseudomonas aeruginosa	enterococcus faecium	No	No	No	No	No	No	No	No	No	No	Resistant to ampicillin (enterococcus), not VRE	Yes	8
213	2	klebsiella oxytoca	escherichia coli	No	Yes	No	No	Yes	No	No	No	No	No	No	Yes	5
214	2	enterococcus faecium	streptococcus parasanguinis	No	No	No	No	No	No	No	No	No	No	No	No	0
215	1	escherichia coli	No	No	No	No	No	No	No	No	No	No	No	Resistant to tmp-smx	Yes	2
216	2	escherichia coli	enterococcus hirae	No	No	No	No	No	No	No	No	No	No	No	Yes	1
217	1	escherichia coli	No	No	Yes	No	No	No	Yes	No	No	No	No	No	Yes	2
218	2	enterococcus faecalis	klebsiella oxytoca	No	No	No	No	No	No	No	No	No	No	No	No	0
